# Effect of a One-Off Educational Session about Enterobiasis on Knowledge, Preventative Practices, and Infection Rates among Schoolchildren in South Korea

**DOI:** 10.1371/journal.pone.0112149

**Published:** 2014-11-05

**Authors:** Dong-Hee Kim, Hak Sun Yu

**Affiliations:** 1 Department of Nursing, College of Nursing, Pusan National University, Yangsan, Gyeongsangnamdo, South Korea; 2 Department of Parasitology, School of Medicine, Pusan National University, Yangsan, Gyeongsangnamdo, South Korea; 3 Immunoregulatory therapeutics group in Brain Busan 21 project, Busan, South Korea; The George Washington University Medical Center, United States of America

## Abstract

Although health education has proven to be cost-effective in slowing the spread of enterobiasis, assessments of the effectiveness of health education to reduce infectious diseases specifically in children are rare. To evaluate the effect of health education on knowledge, preventative practices, and the prevalence of enterobiasis, 319 children from 16 classes were divided into experimental and control groups. Data were collected from May 2012 to March 2013. A 40-minute in-class talk was given once in the experimental group. There were significant differences over the time in the mean scores for children's knowledge of *Enterobius vermicularis* infection in the intervention group compared to the control group (*p*<0.001). After the educational session, the score for knowledge about *E. vermicularis* infection increased from 60.2±2.32 to 92.7±1.19 in the experimental group; this gain was partially lost 3 months later, decreasing to 83.6±1.77 (*p*<0.001). Children's enterobiasis infection prevention practice scores also increased, from 3.23±0.27 to 3.73±0.25, 1 week after the educational session, a gain that was partially lost at 3 months, decreasing to 3.46±0.36 (*p*<0.001). The overall *E. vermicularis* egg detection rate was 4.4%; the rates for each school ranged from 0% to 12.9% at screening. The infection rate at 3 months after the treatment sharply decreased from 12.3% to 0.8% in the experimental group, compared to a decrease from 8.5% to 3.7% in the control group during the same period. We recommend that health education on enterobiasis be provided to children to increase their knowledge about enterobiasis and improve prevention practices.

## Introduction

Although most parasitic infectious diseases have disappeared in developed countries, enterobiasis (pinworm infection) has still often been reported in many developed countries [1]–[3]. In South Korea, the prevalence of total intestinal helminthic parasitic infection rates has sharply decreased from 84.3% in 1971 to 2.4% in 1997 [4], [5]. However, a relatively high egg positive rate of *Enterobius vermicularis* ranging from 4% to 10% has been reported in Korean children during the last decade [6]–[9].

Enterobiasis is transmitted through direct contact with infected (or egg-contaminated) persons or objects. Transmission of *E. vermicularis* commonly occurs by ingesting infectious pinworm eggs. Eggs are transmitted from the anus to the finger, fingernails, or hands when an individual scratches the perianal area where the gravid female worms emerge and deposit eggs. Eggs are spread to underwear and night-clothing and further transmitted to other objects including food and books, desks, and chairs [10]. When dislodged from such objects, the eggs can enter another individual's mouth and nose, thereby being ingested [10], [11]. As a result of this transmission process, children's personal and hygiene habits, such as thumb sucking, overcrowded conditions, and inadequate sanitation, contribute to the spread of enterobiasis in primary schools, where close contact between children occurs [2], [7], [9].

Medication against *E. vermicularis*, such as albendazole, is very effective in treating enterobiasis [12]. However, reinfection is also common in spite of treatment, as the medication only kills the adult worm but not the worm larvae [10]. An important aspect in the failure of single-dose chemotherapy is the continuing presence of infectious eggs in the environment, which facilitate rapid reinfection. Therefore, individuals with enterobiasis require repeated doses of medication to cover the time taken for the eggs to become adult worms. Importantly, most parents in South Korea believe that antihelminthic medications can easily cure every helminthic infection, including those by *E. vermicularis*, by just a one-time treatment [9]. In addition, most kindergarten directors and teachers have limited knowledge of *E. vermicularis* infection [2].

Knowledge of disease has successfully improved many different health outcomes [13]. However, there has been little emphasis on the impact of health education on the prevalence of enterobiasis, despite the incidence of enterobiasis being reduced by encouraging habits of cleanliness in children. Health education promoting knowledge of enterobiasis has proven to be cost-effective in decreasing reinfection rates in schoolchildren [14]. Previously, we evaluated the impact of a health education among pre-school children [2]. We provided brochures on prevention, transmission, and treatment of enterobiasis to parents, as they are in charge of their child's personal hygiene since children younger than 6 years of age are not old enough to be responsible for self-care [2].

In the present study, we conducted an experimental health education session on enterobiasis at primary schools in South Korea and assessed its effect on knowledge about *E. vermicularis* infection, enterobiasis infection prevention practices, and the incidence rate of enterobiasis among primary school children in Korea.

## Subjects and Methods

### Subject recruitment and screening evaluation

Participants were Grade 1 and Grade 2 primary school students (aged 7−9 years) from separate school districts in three distinct regions: an industrial city, an urban site, and a suburban area of South Korea. Recruitment was conducted through the Office of Education websites at each of these sites with a letter informing about the nature, significance, and objectives of the study. Schools were approached with the help of an assistant. Once the assistant had obtained verbal consent from the principal of a school, investigators met with the principal and class teachers of each school to describe the details of the study. The class teachers sent a consent form, a letter of information, and a questionnaire to the parents of each child. A total of 3,840 children from 183 classes in 27 schools underwent a screening for enterobiasis via the sellotape anal swab technique. The parents were each given two pieces of sellotape and written instructions showing how to swab the perianal area of their child with the sellotape and other aspects of the screening procedure. The investigators emphasized that the examinations should be done before the child washed or went to the toilet in the morning to prevent any pinworms eggs from being washed from the area. We cautioned that the chances of making an incorrect diagnosis of enterobiasis increased when the parents did not swab their child's anus first thing in the morning before the child washed. We asked the parents to do this twice, on separate days.

Sample size was determined on the basis of the primary outcome, the score in the knowledge test after education. To have an 80% chance of detecting as significant (at the two sided 5% level) a 10 point difference between the two groups in the knowledge test scores after education, with an assumed standard deviation of 15, the overall sample size required is 74 individuals (37 in each arm of the study). Since this study is cluster-randomized, the sample size had to be larger than if simple randomization had been performed, in order to take into account the design effect. Assuming that the inter-cluster correlation coefficient is 0.1, and a mean cluster size is 21 individuals, the design effect is 3. Therefore, the number of individuals required in each group is 111 (‘Cochrane Consumers and Communication Review Group: cluster randomized controlled trials’. http://cccrg.cochrane.org, March 2014). Assuming the expected drop-out rate of 10%, the final sample size required is 246 (123 in each arm) with a minimum of 6 clusters per arm.

### Study design

**Figure 1:**
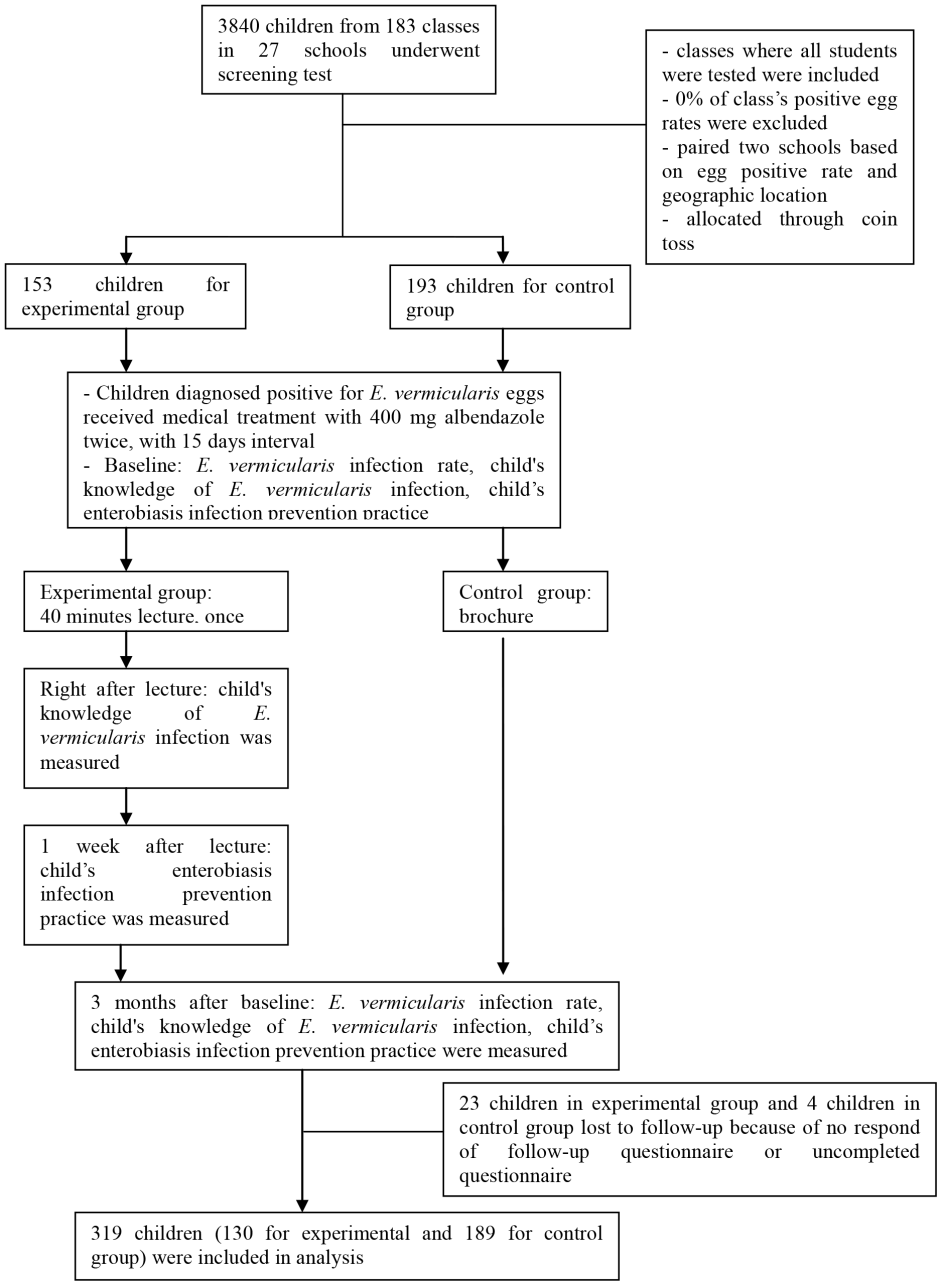
Study design including screening, group allocation, and follow-up. A total of 319 children from 16 classes were invited from among 3,840 children screened from 183 classes. The schools were assigned to either the intervention or the control arms by simple randomization using a coin toss.

The study was designed as a pretest-posttest experiment, with an equivalent control group. We excluded schools that had classes with an incidence rate of 0% at the screening evaluation, and then selected classes in which all students were tested at the screening evaluation. Based on a combination of similar egg positive rates and geographical locations, 10 schools in different regions were involved this study; two schools were in an industrial city, four schools were in an urban site, and four schools were in a suburban area. One or two classes participated at each school. Each school was identified as either an intervention or control group in order to control for the contamination of the control group. The schools were assigned to either the intervention (8 classes from 5 schools) or control (8 classes from 5 schools) arms through a coin toss. A total of 346 children from 16 classes were included at baseline. At post-treatment examination, 319 children (130 for the experimental and 189 for the control group) participated (Fig. 1). Blinding of investigators was not possible as the intervention was educational; however, the investigators were blinded to the exposure status of participants during data collection. In the intervention group, an educational session was given once, for 40 minutes, in a group setting for each class. In the control group, children received an *E. vermicularis* infection brochure. Knowledge of *E. vermicularis* infection and enterobiasis infection prevention practice and the *E. vermicularis* infection rate among children were evaluated at baseline and at 3 months after the intervention. Children's knowledge of *E. vermicularis* infection was assessed on the day of the educational session in order to ensure that their knowledge of *E. vermicularis* infection increased.

### Intervention

The educational program was developed based on the Dick & Carey's Systematic Design of Instruction model [15]. A comprehensive review of the literature, a pilot study, and a focus group interview were used to develop the educational session. The session was comprised of topics such as the lifecycle of *E. vermicularis*, diagnosis of enterobiasis, symptoms and signs, infection and transmission, and treatment and prevention of enterobiasis. A trained teacher provided a 30-minute lecture using visual aids to stimulate interest and support explanations of the educational contents. The lecture included an example situation, describing how one student became infected with a pinworm, and showing how enterobiasis spread to other students in the same classroom. Key messages were reinforced with an interactive quiz, and there was a 10-minute session to answer students' questions.

### Measurement

The main outcome variable is the improvement of 15 or more points in children's knowledge of *E. vermicularis* infection. As there are no currently published scales for children's knowledge on *E. vermicularis* infection and children's preventative practice against enterobiasis infection, the investigators composed a scale, which was validated by experts (including parasitologists, pediatric doctors, internal medicine doctors, school nurses, and schoolteachers). Four pilot studies were conducted to assess comprehension of the questionnaire items. The instrument to measure children's knowledge of *E. vermicularis* infection included 10 dichotomous items (answered as either “correct” [1 point] or “incorrect” [0 points]).

The secondary outcome variables recorded are children's prevention practices and the infection rate 3 months later. The instrument to assess children's prevention practices consisted of 8 Likert-scale items; each Likert item ranged from 1 (never) to 4 (every time).

### Study procedure

Children in both the education and control groups who tested positive for *E. vermicularis* eggs received medical treatment with 400 mg albendazole twice, at a 15-day interval. The pre-treatment structured questionnaire on knowledge of *E. vermicularis* infection was provided to the children by a class teacher. This questionnaire was also provided to the parents of each child, enquiring about demographics and socioeconomic status, the child's enterobiasis infection prevention practices at home, and the parents' knowledge of enterobiasis. Children's knowledge of *E. vermicularis* infection was assessed the day they learned about enterobiasis and 3 months later. One week and three months after the intervention, parents were asked to complete another questionnaire on the children's enterobiasis infection prevention practices at home. *E. vermicularis* egg detection and the reinfection rates were evaluated using a sellotape anal swab 3 months after the intervention.

### Ethics statement

The study was performed after receiving approval from the Ethical Review Committee of Yangsan Pusan National University Hospital, and after informed written consent was obtained from each participant before enrollment. Participation was entirely voluntary. Participants, including principals and teachers of schools, parents, and their children, were free to refuse to participate or withdraw from the study at any time, and were informed that only the aggregate data would be reported. Informed consent for the children in this study was provided by their parents or guardians and by the children. Once we obtained written consent from the parents or guardians to contact their children, the class teacher informed the children that permission to conduct the study had been granted. We briefed the individual children about the study and what they were being asked to do. Children who diagnosed positive for *E. vermicularis* eggs at screening received medical treatment with 400 mg albendazole twice, at a 15-day interval.

### Statistical analysis

We report descriptive statistics for the characteristics of study sites and individual participants. To test the effectiveness of the education program in changing children's knowledge and prevention practices (continuous variables), we used multivariable mixed-effects analysis allowing random effects for clusters and controlling for gender and age. Each estimator was presented with its 95% confidence interval (95% CI). The prevalence of *E. vermicularis* eggs at baseline and after treatment in each group was compared using proportions and the McNemar test.

To examine the effectiveness of the education program in reducing infection (yes: 1; no: 0, binary response), we used multivariable logistic regression allowing random effects for clusters. All statistical analyses were performed using SAS 9.2 (SAS Institute, Inc., Cary, NC) statistical package and *p* values less than 0.05 were considered as statistically significant.

## Results

Participant characteristics were compared between the experimental and control groups, most of which were similar. The small differences observed between groups were not statistically significant. Several characteristics of the participants are shown in Table S1. The overall *E. vermicularis* egg detection rate was 4.4%; the rate for each school ranged from 0% to 12.9% at screening (Table 1). Characteristics of study sites and individual participants are summarized in Table 2. Experimental and control groups enrolled 130 and 189 children, respectively. There were no statistically significant differences in children's baseline characteristics (*p*>0.05).

**Table 1:** Egg positive rates of *E. vermicularis* infection among children in South Korea (n = 3840).

School	No. class	No. examined/total No. student (compliance %)	No. positive (%)
1	2	31/37 (83.8)	4(12.9)
2	11	260/287 (90.6)	21(8.1)
3	6	97/133 (72.9)	7(7.2)
4	2	15/19 (78.9)	1(6.7)
5	6	139/152 (91.4)	9(6.5)
6	6	122/136 (89.7)	7(5.7)
7	4	57/57 (100.0)	3(5.3)
8	13	317/361 (87.8)	16(5.0)
9	6	142/155 (91.6)	7(4.9)
10	10	238/256 (93.0)	11(4.6)
11	9	160/203 (78.8)	7(4.4)
12	6	115/149 (77.2)	5(4.3)
13	8	189/242 (78.1)	8(4.2)
14	10	288/309 (93.2)	12(4.2)
15	9	135/220 (61.4)	5(3.7)
16	10	202/259 (78.0)	7(3.5)
17	8	145/182 (79.7)	5(3.4)
18	8	134/189 (70.9)	4(3.0)
19	12	277/316 (87.7)	8(2.9)
20	9	272/272 (100.0)	8(2.9)
21	4	82/100 (82.0)	2(2.4)
22	6	127/158 (80.4)	3(2.4)
23	4	84/89 (94.4)	2(2.4)
24	7	134/169 (79.3)	1(0.7)
25	2	33/33 (100.0)	0(0.0)
26	2	17/17 (100.0)	0(0.0)
27	3	28/59 (47.5)	0(0.0)
Total	183	3840/4559 (84.2)	163
Mean positive rate			4.4%

**Table 2:** Characteristics of study sites and individual participants (n = 319).

Characteristic		Experimental (n = 130)	Control (n = 189)	P value
Site's characteristic				
Cluster size				
	mean ± SD	19.13±3.94	24.25±2.25	
No. of Conforming Requests/Total				
		11/14(78.6%)	25/26 (96.2%)	
		21/23 (91.3%)	21/21 (100.0%)	
		18/21 (85.7%)	27/27 (100.0%)	
		18/21 (85.7%)	21/21 (100.0%)	
		16/20 (80.0%)	23/24 (95.8%)	
		19/24 (79.2%)	23/24 (95.8%)	
		14/16 (87.5%)	26/26 (100.0%)	
		13/14 (92.9%)	23/25 (92.0%)	
Total		130/153 (85.0%)	189/194 (97.4%)	
Children's characteristics				
Sex				
	Male	69 (53.9%)	104 (57.1%)	0.581
	Female	59 (46.1%)	78 (42.9%)	
Age				
	Mean ± SD	8.22±0.70	8.24±0.67	0.827
House type				
	Apartment	109 (85.2%)	158 (86.8%)	0.823
	Non-apartment	19 (14.8%)	24 (13.2%)	
Job of parents				
	Single	71 (55.5%)	99 (54.4%)	0.261
	Both	57 (44.5%)	83 (45.6%)	
Family size				
	≤3 persons	96 (76.8%)	142 (78.5%)	0.583
	>4 persons	29 (23.2%)	39 (21.5%)	

Between-group comparisons of knowledge of and prevention practices for *E. vermicularis* infection, as well as the prevalence of *E. vermicularis* egg positive rates, are show in Table 3. There were significant time effects in the mean scores for children's knowledge of *E. vermicularis* infection in the intervention group compared to the control group (*p*<0.001). Regarding *E. vermicularis* infection prevention practices, the experimental group increased from 3.22 to 3.45 (a difference of 0.23), whereas the control group increased from 3.19 to 3.23 (a difference of 0.04) between baseline and 3 months after treatment (*p*<0.001). The experimental group had a higher increase in knowledge test scores than the control group (adjusted difference  = 1.95 [95% CI, 1.57–2.34]; *p*<0.001). The experimental group also had a higher increase in prevention practices than the control group (adjusted difference  = 0.19 [95% CI, 0.13–0.25]; *p*<0.001). Clustering was considered in all logistic and multivariate regression models.

**Table 3:** Comparison of the prevalence of *E. vermicularis* egg positive rates, knowledge, and prevention practices for *E. vermicularis* infection between groups (n = 319).

	Time	Experimental (n = 130)	Control (n = 189)	P value of Difference	Treatment Difference
		n (%)/mean ± SD	n (%)/mean ± SD		(95% CI)
Knowledge					
Baseline		6.02±2.32	6.12±2.09		
3 months after		8.36±1.77	6.45±2.04		
Difference		2.35±2.43	0.33±0.97	<0.001	1.96‡ (1.57–2.34)
Preventing Practice					
Baseline		3.22±0.28	3.19±0.43		
3 months after		3.45±0.36	3.23±0.40		
Difference		0.23±0.37	0.04±0.18	<0.001	0.19‡ (0.13–0.25)
Infection rate					
Baseline	Positive	16(12.3%)	16 (8.5%)	0.263	
3 months after	Positive	1(0.8%)*	7 (3.7%)*	0.175	0.20†(0.02–2.41)
Baseline - 3 months after	Positive – positive	0(0.0%)	3(1.6%)		
	(re-infection case)				
	Negative – positive	1(0.8%)	4(2.1%)		
	(New infected case)				

*Statistically significant between baseline and 3 months in experimental group (p<0.001) and control group (*p* = 0.049), based on the McNemar test.

† OR was adjusted for clusters as a random effect.

‡ Mean difference was adjusted for clusters as a random effect, as well as gender and age.

The incidence rate was lower in the experimental group, although this finding was not significant after adjustment for the clusters as random effects (adjusted odds ratio  = 0.20, 95% CI  = 0.02–2.41, *p* = 0.175). The infection rate at 3 months after treatment sharply decreased from 12.3% to 0.8% in the experimental group (*p*<0.001), while that in the control group decreased from 8.5% to 3.7% (*p* = 0.049) during the same period. Some children were diagnosed with new infections at 3 months after treatment; however, the number of new infections in the experimental group was lower than that in the control group. Moreover, although children who tested positive for *E. vermicularis* eggs were treated with antihelminthic drugs at baseline, *E. vermicularis* reinfection was observed in the control group.

We also jointly compared baseline, post-education, and 3-month changes in the experimental group. Correct answer rates on the *E. vermicularis* infection knowledge test in the experimental group are shown in Table 4. After the educational session, the score for knowledge about *E. vermicularis* infection increased from 60.2±2.32 to 92.7±1.19 in the experimental group; this gain was partially lost 3 months after the educational session, decreasing to 83.6±1.77 (*p*<0.001). The correct answer rate was 34.6% to 78.5% at baseline, 80.0% to 99.2% at post-education, and 75.4% to 93.1% 3 months after the intervention. At baseline, the item “Proper teeth brushing can prevent *E. vermicularis* infection” (34.6%) had the lowest rate of correct answers, followed by “Weekly change of underwear is important for preventing *E. vermicularis* infection” (36.9%), “*E. vermicularis* is not transmitted to other humans via hand contact” (47.7%), and “*E. vermicularis* infection can be treated by taking antihelminthic medication once” (50.0%); the rates for these items increased to over 80% correct responses at post-education, and over 75% correct 3 months later.

**Table 4:** Assessment of children's correct answer rates on *E. vermicularis* infection knowledge test (Experimental group: n = 130).

Items	Correct answer rate (%)		
	Baseline	After education	3 month after
*E. vermicularis* is parasitic worm that can live inside the human	74.6	99.2	88.5
*E. vermicularis* is not transmitted to other human via hands	47.7	86.9	88.5
*Enterobius vermicularis* infection can be diagnosed by using sellotape anal technique	68.5	98.5	83.1
Child with *E. vermicularis* may have anal itching	71.5	98.5	93.1
The habits of sucking fingers or biting nails is associated with *E. vermicularis* infection	65.4	93.1	89.2
Good hand hygiene can help prevent the spread of *E. vermicularis* infection	78.5	95.4	91.5
Proper brushing teeth can be preventive *E. vermicularis* infection	34.6	91.5	79.2
Anal cleansing can help prevent *E. vermicularis* infection	73.8	95.4	86.2
Weekly change of underwear is good for preventing *E. vermicularis* infection	36.9	80.0	75.4
*E. vermicularis* infection can be treated by taking antihelminthic medication once	50.0	88.5	80.0
Over all M± SD	60.2±2.32	92.7±1.19	83.6±1.77
Generalized linear mixed model test statistic (*p*) 157.230 (<0.001)			

Results for children's practices for the prevention of *E. vermicularis* infection are shown in Table 5. Children's enterobiasis infection prevention practice scores also increased, from 3.23±0.27 to 3.73±0.25, 1 week after the educational session, and then partially decreased to 3.46±0.36 after 3 months (*p*<0.001). Items related to hand washing had lower scores than other items, such as keeping nails short and cleaning underwear. After 3 months, the item “My child does not bite his/her nails” had the lowest score of all items at both 1 week and 3 months after the health education session.

**Table 5:** Children's prevention practices for *E. vermicularis* infection (Experimental group: n = 130).

Items	Baseline	1 week after	3 month after
My child practices hand washing after defecation	3.00±0.29	3.71±0.47	3.32±0.60
My child practices hand washing before eating	2.72±0.51	3.57±0.53	3.32±0.61
My child practices hand washing after coming in from outside	2.89±0.44	3.77±0.48	3.51±0.59
My child does not sucking fingers or toys	3.30±0.86	3.68±0.62	3.50±0.79
My child does not biting nails	3.12±0.97	3.45±0.82	3.28±0.96
My child keeps the nails short	3.58±0.75	3.78±0.54	3.40±0.55
My child practices proper anal cleansing	3.49±0.61	3.96±0.20	3.59±0.55
My child wears clean underwear	3.63±0.60	3.96±0.20	3.65±0.48
Over all M± SD	3.23±0.27	3.73±0.25	3.46±0.36
Generalized linear mixed model test statistic (*p*)		149.486 (<0.001)	

## Discussion

Mass drug administration is the most effective means to control enterobiasis, but this method also has some limitations in that it does not prevent reinfections. In a previous study, we found new infection cases in the mass drug administration treatment group at both 3 and 6 months after treatment [2]. Moreover, reinfection increases the financial burden placed on preventative medicine programs. Consensus among government health employees and social workers might be necessary, because part of the cost of group treatment must be covered by the government. Furthermore, there is concern that mass drug administration might lead to the development of drug-resistant parasites [16]. The development of drug resistance in nematodes that infect humans is considered inevitable, given the number of species infecting livestock that are now resistant to antihelminthic agents due to continuous and extensive drug use [17], [18]. Strategies to reduce the overall incidence of enterobiasis infection are likely to require an integrated approach, including pharmacological treatment to reduce the infection rate and health education for prevention and sustainable control.

Our results showed that health-education increased students' knowledge about enterobiasis transmission and changed their behavior. Notably, students washed their hands more frequently and sucked their fingers or toys less after the health education session (Tables 4 and 5). Most instances of infection transmission could be effectively prevented by repeated hand washes; therefore, behavioral changes might contribute to a reduction in enterobiasis rates. Interestingly, most students remembered general facts about enterobiasis (life cycle, transmission route, prevention, etc.) 3 months after receiving an educational lecture (Table 4).

In Korea, over 80% of people were infected with intestinal helminthic parasites in the 1960s [19]. Most people at the time lived in poor environments in which parasitic infections were easily transmitted [20]. Additionally, farmers at the time used “Nightsoil” (human feces and urine) as fertilizer on food crops. Furthermore, underground water was easily contaminated by parasite eggs found in human and animal feces; therefore, parasites were not easily eradicated in humans by mass drug administration. However, after the South Korean government launched a life environmental improvement project (“Saemaeul” [new village] Movement) and established the Korean Parasite Eradiation Association (KPEA), parasitic infection rates rapidly decreased. The Saemaeul Movement improved the drinking water supply system and sewage treatment system. The KPEA (now, Korea Association of Health Promotion) strived to eradicate parasite infections by conducting periodic examinations of the parasite infection rate, treating infected people, and providing preventative education for inhabitants in endemic areas. After the 1990s, most intestinal parasitic infection rates decreased to less than 3.0% in South Korea, with soil-transmitted helminthic infection rates decreasing to less than 1.0% [2], [9].

However, in spite of the struggles of the government, enterobiasis has yet to be eliminated. One of the reasons for this is that there are misconceptions about enterobiasis in South Korea [2], [9]. The first is the belief that parasitic infections, including enterobiasis, have already disappeared in South Korea; due to this belief, approximately half of children have not taken medication against helminthic parasites, including pinworms. The second misconception is that enterobiasis can easily be cured by a one-time anti-helminthic medication, as is the case for other intestinal nematodes. It was recently shown that the numbers of young children being cared for in group facilities, including private educational institutes, have increased, and the employees and teachers of these facilities have such misconceptions [2]. Therefore, opportunities for infection and transmission from child to child have increased as a result. These misconceptions should be rectified through health education providing the correct information, which would be expected to result in a rapid decrease in infection rates [2], [9].

Visual educational materials targeting schoolchildren have been shown to have a positive effect on knowledge and attitudes [18], [21], [22]. In this study, the educational information on enterobiasis included cartoon materials and real-life visual representations, such as microscopic images of pinworms, sellotape used for the diagnosis of enterobiasis, and the medications used to treat it. Most students who participated in this study were interested and immersed in the example situation as if he/she was the infected student. We believe that storytelling using cartoon materials was effective in helping children to focus on the educational contents. Moreover, descriptions of other aspects, such as the sellotape anal swab technique for diagnosis, elicited a response from the students, as they had themselves experienced this during the baseline data collection period. Finally, the interactive quiz emphasized the major educational content. This interactive health education session might lead to behavioral changes that result in decreasing the risk of *E. vermicularis* infection.

The health education session increased knowledge about enterobiasis. We were worried that at the 3-month follow-up assessment children would not remember the information acquired earlier. However, interestingly, the majority of children remembered most of the information they had learned on the subject. In addition, their prevention practices against *E. vermicularis* infection were maintained in their daily lives. Some children were administered the antihelminthic drug (albendazole) at the same time as their family members (personal communication), indicating that educating children may also have an indirect influence on their family members. These results could provide substantial gains in the elimination of enterobiasis in Korea, since Grade 1 and 2 primary school students are the most commonly infected population [6], [23].

The prevalence of the *E. vermicularis* egg positive rate was not significant after adjusting for clusters as a random effect in this study, although the infection rates in the experimental group showed larger changes than in the control group. In a previous study, Gai et al. reported the negative relationship between the rate of parasitic infection and knowledge of prevention [24]. Enterobiasis was successfully treated with an anthelminthic agent. Children in both the education and control groups who tested positive for *E. vermicularis* eggs received medical treatment with albendazole at baseline according to ethical considerations. Moreover, we evaluated the effect of the intervention for 3 months since participants changed classes as they advanced into the next grade. Future long-term evaluation studies need to assess whether health education increasing students' knowledge about enterobiasis transmission impacts infection rates.

Our study has a few limitations. First, there was potential confounding effect due to interaction between teachers in the experimental and control groups, despite our attempts to maintain a distance between groups by selecting them from different districts. Second, we asked the parents to assess their children's enterobiasis infection prevention practices, that is an indirect measure and might not be accurate. In addition, we asked the parents to do the Sellotape swab of their child's anus first thing in the morning, before the child had washed. There is a possibility that the children's parents decided to wash their child first, before preparing the swab, to show that their child had not become reinfected. This would influence the infection rates 3 months after the educational session. Third, as the study could not be double-blinded, other factors may have affected the results. Furthermore, it was not clear whether the educational session would have an impact on infection rates due to lack of previous studies. That is why we tested children's knowledge as a primary outcome. To detect the infection rate difference of 2.9 derived in this study, the total number of clusters required is 117. Due to the lack of statistical power, the result regarding infection rates need to be interpreted with caution and future studies with large sample sizes are required.

In spite of these limitations, we believe that health education can be a cost-effective and safe strategy to decrease enterobiasis and other childhood diseases through to adulthood, as behaviors obtained early in life can result in long-term favorable sanitary habits later in life.

## Supporting Information

Table S1 **Distribution of baseline characteristics of study participants.**
(DOC)

## References

[1] Degerli S, Malatyali E, Ozcelik S, Celiksoz A (2009) Enterobiosis in Sivas, Turkey from past to present, effects on primary school children and potential risk factors. Turkiye Parazitol Derg 33: 95–100. 19367557

[2] Kang IS, Kim DH, An HG, Son HM, Cho MK, et al (2012) Impact of health education on the prevalence of enterobiasis in Korean preschool students. Acta Trop 122: 59–63. 22172595 10.1016/j.actatropica.2011.11.017

[3] Bager P, Vinkel Hansen A, Wohlfahrt J, Melbye M (2012) Helminth infection does not reduce risk for chronic inflammatory disease in a population-based cohort study. Gastroenterology 142: 55–62. 21983081 10.1053/j.gastro.2011.09.046

[4] Report of Ministry of Health and Welfare of Republic of Korea and the Korea Association of Health (1971) Prevalence of intestinal parasitic infections in Korea—1st report. Ministry of Health and Welfare of Republic of Korea and the Korea Association of Health.

[5] Report of Ministry of Health and Welfare of Republic of Korea and the Korea Association of Health (1997) Prevalence of intestinal parasitic infections in Korea -6th report. Ministry of Health and Welfare of Republic of Korea and the Korea Association of Health.

[6] Kim BJ, Lee BY, Chung HK, Lee YS, Lee KH, et al (2003) Egg positive rate of Enterobius vermicularis of primary school children in Geoje island. The Korean J Parasitol 41: 75–77. 12666734 10.3347/kjp.2003.41.1.75 PMC2717486

[7] Song HJ, Cho CH, Kim JS, Choi MH, Hong ST (2003) Prevalence and risk factors for enterobiasis among preschool children in a metropolitan city in Korea. Parasitol Res 91: 46–50. 12884012 10.1007/s00436-003-0836-3

[8] Kang S, Jeon HK, Eom KS, Park JK (2006) Egg positive rate of Enterobius vermicularis among preschool children in Cheongju, Chungcheongbuk-do, Korea. Korean J Parasitol 44: 247–249. 16969064 10.3347/kjp.2006.44.3.247 PMC2532658

[9] Kim DH, Son HM, Kim JY, Cho MK, Park MK, et al (2010) Parents' knowledge about enterobiasis might be one of the most important risk factors for enterobiasis in children. Korean J Parasitol 48: 121–126. 20585527 10.3347/kjp.2010.48.2.121 PMC2892566

[10] Roberts LS, Schmidt GD, Janovy J (2009) Foundations of parasitology. Boston: McGraw-Hill Higher Education. xvii, 701 p.

[11] Cook GC (1994) Enterobius vermicularis infection. Gut 35: 1159–1162. 7959218 10.1136/gut.35.9.1159 PMC1375686

[12] St Georgiev V (2001) Chemotherapy of enterobiasis (oxyuriasis). Expert Opin pharmacother 2: 267–275. 11336585 10.1517/14656566.2.2.267

[13] Owais A, Hanif B, Siddiqui AR, Agha A, Zaidi AK (2011) Does improving maternal knowledge of vaccines impact infant immunization rates? A community-based randomized-controlled trial in Karachi, Pakistan. BMC Public Health 11: 239. 21496343 10.1186/1471-2458-11-239 PMC3094245

[14] Nithikathkul C, Akarachantachote N, Wannapinyosheep S, Pumdonming W, Brodsky M, et al (2005) Impact of health educational programmes on the prevalence of enterobiasis in schoolchildren in Thailand. J Helminthol 79: 61–65. 15831115 10.1079/joh2004272

[15] Dick W, Carey L, Carey JO (2005) The systematic design of instruction. Boston; London: Pearson/Allyn & Bacon. xx, 376 p.

[16] Keiser J, Utzinger J (2010) The drugs we have and the drugs we need against major helminth infections. Adv Parasitol 73: 197–230. 20627144 10.1016/S0065-308X(10)73008-6

[17] Albonico M (2003) Methods to sustain drug efficacy in helminth control programmes. Acta Trop 86: 233–242. 12745140 10.1016/s0001-706x(03)00043-3

[18] Bieri FA, Gray DJ, Williams GM, Raso G, Li YS, et al (2013) Health-education package to prevent worm infections in Chinese schoolchildren. N Engl J Med 368: 1603–1612. 23614586 10.1056/NEJMoa1204885

[19] Seo BS, Rim HJ, Loh IK, Lee SH, Cho SY, et al (1969) [Study On The Status Of Helminthic Infections In Koreans]. Kisaengchunghak chapchi 7: 53–70. 12913542 10.3347/kjp.1969.7.1.53

[20] Kim CH, Park CH, Kim HJ, Chun HB, Min HK, et al (1971) [Prevalence Of Intestinal Parasites In Korea]. Kisaengchunghak chapchi 9: 25–38. 12913622 10.3347/kjp.1971.9.1.25

[21] Myint UA, Bull S, Greenwood GL, Patterson J, Rietmeijer CA, et al (2010) Safe in the city: developing an effective video-based intervention for STD clinic waiting rooms. Health Promot Pract 11: 408–417. 18544663 10.1177/1524839908318830

[22] Naldi L, Chatenoud L, Bertuccio P, Zinetti C, Di Landro A, et al (2007) Improving sun-protection behavior among children: results of a cluster-randomized trial in Italian elementary schools. The “SoleSi SoleNo-GISED” Project. J Invest Dermatol 127: 1871–1877. 17460732 10.1038/sj.jid.5700835

[23] Lee KJ, Ahn YK, Ryang YS (2001) *Enterobius vermicularis* egg positive rates in primary school children in Gangwon-do (province), Korea. Korean J Parasitol 39: 327–328. 11775335 10.3347/kjp.2001.39.4.327 PMC2721220

[24] Gai L, Ma X, Fu Y, Huang D (1995) [Relationship between the rate of parasitic infection and the knowledge of prevention]. Zhongguo ji sheng chong xue yu ji sheng chong bing za zhi 13: 269–272. 8732079

