# Supplementary material for: Effect of a One-Off Educational Session about Enterobiasis on Knowledge, Preventative Practices, and Infection Rates among Schoolchildren in South Korea
Source: PLoS One. 2014 Nov 5;9(11):e112149. doi: 10.1371/journal.pone.0112149 (PMC4221566; doi:10.1371/journal.pone.0112149)
Supplement: Table S1 — Distribution of baseline characteristics of study participants. (DOC) [file pone.0112149.s001.doc]

Supplementary Table 1. Distribution of baseline characteristics of study participants (n=319)

| Variable | Experimental | Control | X^2^ / t-test (p) |
| --- | --- | --- | --- |
|  | N(%)/M±SD | N(%)/M±SD |  |
| Gender  Boys  Girls  Subtotal | 69(53.9)  59(46.1)  128 | 104(57.1)  78(42.9)  182 | 0.319(.572) |
| Age (years)  7  8  9  Subtotal | 20(15.5)  60(46.5)  49(38.0)  129 | 25(13.4)  91(48.9)  70(37.6)  186 | 0.322(.851) |
| Experience of parasite infection  Yes  No  No idea  Subtotal | 4(3.1)  111(86.7)  13(10.2)  128 | 6(3.3)  159(87.4)  17(9.3)  182 | 0.062(.969) |
| Child’s premedication with anthelmintics  Yes  No  Subtotal | 81(63.3)  47(36.7)  128 | 113(63.5)  65(36.5)  178 | 0.001(.971) |
| Having a job  Both parent  One  Subtotal | 57(44.5)  71(55.5)  128 | 83(45.6)  99(54.4)  182 | 0.035(.852) |
| Family size  ≤3  ≥4  Subtotal | 96(76.6)  29(23.2)  125 | 142(78.5)  39(21.5)  181 | 0.117(.732) |
| Number of siblings  1  2  ≥3  Subtotal | 21(16.4)  83(64.8)  24(18.8)  128 | 26(14.3)  127(69.8)  29(15.9)  182 | 0.842(.656) |
| Housing  Apartment  Private residence  Subtotal | 109(85.2)  19(14.8)  128 | 158(86.8)  24(13.2)  182 | 0.173(.678) |
| Family’s premedication with anthelmintics  Yes  No  Subtotal | 70(86.4)  11(13.6)  81 | 95(90.5)  10(9.5)  105 | 0.751(.386) |
| Parent’s experience of education about enterobiasis  Yes  No  Subtotal | 21(16.4)  107(83.6)  128 | 18(10.1)  160(89.9)  178 | 2.652(.103) |
| Parent’s knowledge of enterobiasis (M±SD)  Subtotal | 8.19±2.11  128 | 8.25±1.86  178 | .286*(.775) |

*Independent t-test
